# Recent clinical trends in Toll‐like receptor targeting therapeutics

**DOI:** 10.1002/med.21553

**Published:** 2018-11-18

**Authors:** Muhammad Ayaz Anwar, Masaud Shah, Jason Kim, Sangdun Choi

**Affiliations:** ^1^ Department of Molecular Science and Technology Ajou University Suwon Korea; ^2^ J2H Biotech Suwon Korea

**Keywords:** adjuvant, clinical trial, drug, innate immunity, Toll‐like receptor

## Abstract

Toll‐like receptors (TLRs) are germline‐encoded receptors that are central to innate and adaptive immune responses. Owing to their vital role in inflammation, TLRs are rational targets in clinics; thus, many ligands and biologics have been reported to overcome the progression of various inflammatory and malignant conditions and support the immune system. For each TLR, at least one, and often many, drug formulations are being evaluated. Ligands reported as stand‐alone drugs may also be reported based on their use in combinatorial therapeutics as adjuvants. Despite their profound efficacy in TLR‐modulation in preclinical studies, multiple drugs have been terminated at different stages of clinical trials. Here, TLR modulating drugs that have been evaluated in clinical trials are discussed, along with their mode of action, suggestive failure reasons, and ways to improve the clinical outcomes. This review presents recent advances in TLR‐targeting drugs and provides directions for more successful immune system manipulation.

## 1 INTRODUCTION

Toll‐like receptors (TLRs) are integral membrane bound receptors that are vital for innate immunity and help to shape the adaptive immune response. These receptors are triggered by a variety of pathogen‐associated molecular patterns (PAMPs), and danger‐associated molecular patterns (DAMPs). PAMPs are parts of pathogens such as lipoproteins, lipopeptides, and flagella, as well as nucleic acids (either single‐stranded or double‐stranded DNA or RNA),^1^ while DAMPs are self‐molecules that include multiple heat shock proteins, S100, and high mobility group box 1 (HMGB1) that are released in response to injury or any other anomaly in the cells.^2^, ^3^ DAMPs or PAMPs can engage a variety of TLRs; those situated on the cell surface primarily engage TLRs that function on the cell membrane, whereas intracellularly localized TLRs are activated by nucleic acid components, which are made available after pathogen endocytosis or replication. Given their functions, TLRs are considered as the first line in immune defense.^1^, ^4^

The number of functional TLRs can vary in mammals; however, they all have conserved functions, namely the activation of inflammatory mediators. Humans have 10 TLRs; TLR2 (can heterodimerize with TLR1 or TLR6), TLR4, TLR5, and TLR10 are present at the cell membrane, whereas TLR3, TLR7, TLR8, and TLR9 are functionally localized to endosomes (Table 1).^1^, ^5^, ^6^ These receptors invariably work as homodimers or heterodimers, and several studies have suggested that they exhibit unusual dimer characteristics.^7^, ^8^ All TLRs form homo‐ and heterodimers, except for TLR3 and TLR5, which are currently considered strictly homodimeric, in the absence of empirical evidence to the contrary.

**Table 1:** Natural and synthetic ligands for TLRs

TLR	Expression	PAMPs	DAMPs	Nature of molecules investigated in clinical trials
TLR2 (1/6)	B, Mo, Mac, DCs, Plt N, MyDCs, Mc	Lipoproteins, zymosan, peptidoglycan	HSPs, HMGB1, hyaluronan, HDL (modified)	Lipopeptide, recombinant protein, antibody
TLR3	DC, B, Plt	Viral dsRNA	Self dsRNA	Polyinosinic‐polycytidylic acid (polyIC, poly‐ICLC, polyIC12U), anti‐TLR3 antibody
TLR4	Mo, Mac, N, MyDCs, Mc, B, IE, Plt	Lipopolysaccharide	HSPs, fibrinogen, heparin sulfate, fibronectin, HA, HMGB1, hyaluronan, oxidized LDL, ANG II	Lipid A derivates (glycolipids), anti‐TLR4 antibody, polysaccharide
TLR5	Mo, Mac, DC, IE	Flagellin	HMGB1	Flagella and flagella based molecules
TLR7	Mo, Mac, pDC, B, Plt	Viral ssRNA	Self ssRNA	SM
TLR8	Mo, Mac, DC, Mc	Viral ssRNA	Self ssRNA	SM
TLR9	Mo, Mac, pDC, B, Plt	Bacterial and viral CpG DNA	Self DNA	DNA based, synthetic ssDNA molecules
TLR10	LN, Mo, S, B, L	NA	NA	NA

Abbreviations: ANG II, angiotensin II; B, B cell; DAPM, danger‐associated molecular pattern; DC, dendritic cell; dsRNA, double‐stranded RNA; HA, hyaluronic acid; HDL, high‐density lipoprotein; HMGB1, high mobility group box 1; HSPs, heat shock proteins; IE, intestinal epithelium; L, lung; LC, liver cell; LDL, low‐density lipoprotein; LN, lymph node; Mac, macrophage; Mc, mast cell; Mo, monocyt; MyDC, myeloid dendritic cell; N, neutrophil; NA, not available; PAMP, pathogen‐associated molecular pattern; pDC, plasmacytoid DC; Plt, platelet; S, spleen; SM, small molecule; ssRNA, single‐stranded RNA; TLR, Toll‐like receptor.

**Figure 1:**
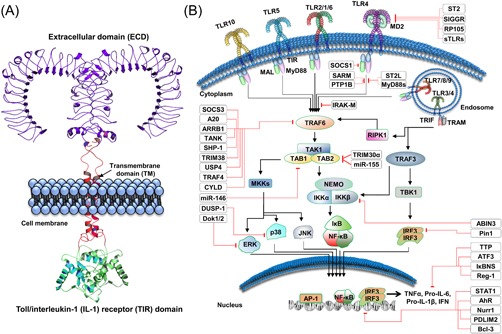
Generalized structure and signaling mechanisms of Toll‐like receptors. A, A typical TLR is composed of three distinct domains, an ECD, a TM domain, and a TIR domain. B, Conventionally, TLRs are divided into two categories; cell surface functional, and endosomally functional TLRs. Endosomal TLRs are mainly activated through nucleic acids, while cell surface–expressed TLRs are activated by a variety of ligands, including proteins and lipoproteins. Upon sensing PAMPs or DAMPs, TLRs dimerize and reorient their TIR domains, allowing the docking of intracellularly localized TIR‐containing proteins, including MAL, MyD88, TRIF, and TRAM. The majority of TLRs convey downstream signals through MyD88; however, TLR3 can signal only through TRIF. Exceptionally, TLR4 can transmit signals through both the MyD88 and TRIF adapter proteins. Therapeutics targeting immune‐related diseases mediated by TLRs are reported to modulate these signaling mechanisms. There are many internal mechanisms that come into action to regulate TLR‐mediated inflammation. These act at all levels starting from cell surface interaction to dent the TLR dimerization, and cytoplasmic interactions to block adapter molecules, alter the posttranslational modification state, and finally in the nucleus to counter overexpression of various interleukins and cytokines. There are many microRNAs that reduce the mRNA stability of different cytokines. All these mechanisms ensure a balanced response toward the invading pathogen or DAMP that unbalance the homeostasis. ABIN3, A20 binding and inhibitor of NFκB‐3; AhR, aryl hydrocarbon receptor; AP1, activated protein 1; ATF3, activating transcription factor 3; Bcl‐3, B‐cell lymphoma 3‐encoded protein; CYLD, cylindromatosis; DOK, downstream of tyrosine kinases; DUSP, dual specificity phosphatases; ECD, extracellular domain; ERK, extracellular‐regulated kinase; IFN, interferon; IkB, inhibitor of κB; IKK, inhibitor of κB kinase; IL, interleukin; IRF, interferon response factor; IRAK‐M, interleukin receptor‐associated kinase M; JNK, c‐Jun N‐terminal kinase; MAL, MyD88 adapter like; MD2, myeloid differentiation factor 2; MIR, microRNA; MKK, mitogen‐activated protein kinase kinase; mRNA, messenger RNA; MyD88, myeloid differentiation primary response 88; MyD88s, myeloid differentiation primary response 88 short; NEMO, NF‐κB essential modulator; NFKBID, NF‐κB inhibitor δ; NF‐κB, nuclear factor κB; Nurr1, nuclear receptor related 1 protein; p38, protein 38; PDLIM2, PDZ and LIM domain 2; PTP1B, protein tyrosine phosphatase‐1B; Reg‐1, regnase‐1; RIPK‐1, receptor interacting protein kinase 1; RP105, radioprotective 105 kDa protein; SARM, sterile α and armadillo‐motif containing protein; SHP‐1, Src homology region 2 domain‐containing phosphatase‐1; SIGGR, single immunoglobulin IL1R‐related molecule; SOCS, suppressor of cytokine signaling; ST2, suppression of tumorigenity 2; ST2L, membrane bound ST2; STAT, signal transducers and activators of transcription; sTLRs, soluble Toll‐like receptor; TAB, TAK‐1‐binding protein; TAK1, transforming growth factor β‐activated kinase 1; TANK, TRAF‐associated NF‐κB activator; TBK1, TANK‐binding kinase 1; TIR, Toll/interleukin‐1 receptor; TLR, Toll‐like receptor; TM, transmembrane domain; TNF‐α, tumor necrosis factor α; TRAF, tumor necrosis factor receptor (TNF‐R)‐associated factor; TRAM, TRIF‐related adapter molecule; TRIF, TIR‐domain‐containing adapter‐inducing interferon‐β; TRIM38, tripartite motif 38; TTP, tristetraprolin; USP4, ubiquitin‐specific protease 4 [Color figure can be viewed at wileyonlinelibrary.com]

TLRs are composed of three distinct domains, an extracellular domain (ECD) that senses the ligand, a transmembrane domain (TM) that anchors the TLR within membranes, and Toll/interleukin‐1 (IL‐1) receptor (TIR) domain that interacts with other TIR‐containing adapters to initiate signaling (Figure 1A).^9^, ^10^, ^11^ TLRs signaling depends on its dimer assembly, and in the absence of any ligand, TLRs exist either in monomeric forms or weakly dimeric forms that are unable to initiate the signaling. While ligand binding confers dimer stability and induces a conformational change that reorients the TIR domain and initiates signaling.^12^, ^13^ TLRs are ideal targets for immune modulation strategies, since they have both known modulators and proven therapeutic potential (Table 1).

Therefore, it is rational to harness their potential for improving vaccines efficacy, to treat cancers (breast and bladder cancers^14^, ^15^), to inhibit their activity in inflammatory diseases (for instance; rheumatoid arthritis (RA)^16^ and multiple sclerosis^17^), to modulate them in autoimmune diseases such as systemic lupus erythematosus (SLE),^18^ to fine tune them to generate specific responses (humoral vs cell‐mediated immune responses) and to curb the menopausal osteoporosis.^19^, ^20^, ^21^ Other than these, there are numerous diseases where TLRs play important roles, for that, the interested reviewers are encouraged to consult recent reviews.^22^, ^23^, ^24^ Given their extensive potential benefits, they are the target‐of‐choice for many therapeutic endeavors, and these efforts are bearing fruit, with many compounds that target TLRs are currently at various stages of evaluation in clinical trials.^19^

We have therefore revised and gathered relevant data and wish to present it to the scientific community to guide them in their future investigations. The data in this paper has been collected from ongoing clinical trials that either target TLRs or use them to induce improved responses. Data have been organized in a reader‐friendly manner, focusing on the clinical condition, the type of TLR being targeted, the failures and successes of drugs in different phases of clinical trials, and the synergistic efficacy of TLR ligands as adjuvants.

## 2 TLR SIGNALING

In induction of inflammatory responses, TLRs primarily act via the myeloid differentiation response protein 88 (MyD88) and TIR‐domain‐containing adapter‐inducing interferon‐β (TRIF)–mediated pathways.^25^, ^26^, ^27^, ^28^ On sensing PAMPs or DAMPs, TLRs dimerize and reorient their TIR domains, which allow docking of the TIR containing proteins, MyD88 and MyD88 adapter‐like (MAL). MAL is a bridging adapter frequently involved in TLR4, and to a lesser extent in TLR2, signaling pathways, and it interacts with MyD88 through TIR‐TIR interaction. In addition to the TIR domain, MyD88 contains a death domain that facilitates its interaction with interleukin‐1 receptor (IL1R)‐associated kinase 4 (IRAK4), which can both auto‐phosphorylate and trans‐phosphorylate IRAK2/1.^29^ This inter‐domain interaction results in a large multimeric molecule, referred to as myddosome, the phosphorylation of which leads to the activation and dimerization of tumor necrosis factor (TNF) receptor–associated factor 6 (TRAF6).^30^ TRAF6, an E3 ligase that is activated via autoubiquitination in an sequestosome 1 (SQSTM1/p62)‐dependent manner, mediates the ubiquitination of transforming growth factor‐β–activated kinase 1 (TAK1).^31^, ^32^ TAK1 belongs to the mitogen‐activated protein kinase kinase kinase (MAPKKK) family and forms a complex with the TAK1 binding proteins, TAB1–3. TAK1 deficiency reduces inflammatory signaling across TLRs; however, no such difference has been observed in response to a deficiency of TAB proteins.^33^, ^34^ TAK1/TABs signaling then branches into two arms: activation of nuclear factor κB (NF‐κB) and MAPK. NF‐κB is held inactive in the cytoplasm by inhibitor of κB (IκB), which is phosphorylated by IκB kinase α (IKKα) and IKKβ, and degraded via ubiquitin mediated‐proteasomal degradation, exposing a nuclear localization signal in NF‐κB, and subsequently translocating to the nucleus as reviewed by Kawai and Akira.^35^ NF‐κB is a hub molecule for inflammatory signals and it induces the expression of a wide array of molecules that cause inflammation, alteration in cell surface receptors, expression of pro‐ and anti‐cancerous molecules, and perturbation in cell motility, among other responses. TAK1 also activates MAPK family members, including MKK7 and/or MKK6/3, resulting in the phosphorylation of p38 and JNK, and culminating in the activation of activated protein 1 (AP1) family transcription factors and messenger RNA (mRNA) stabilization of various genes involved in the regulation of inflammation (Figure 1B).^4^, ^36^

TRIF–dependent signaling is a separate arm of TLR signaling perpetuated only by TLR3 and TLR4, where TRIF interacts with TRAF3 and TRAF6. TRAF6 interacts with receptor interacting protein kinase 1 (RIPK‐1), which transduces the signal by activating TAK1, a crucial branch point in the TLR signaling pathway. TRAF3 activates IKK‐related kinases, such as TANK‐binding kinase 1 (TBK1) and IKKi, along with NEMO, and the transduced signals culminate in interferon (IFN)‐regulatory factor 3 (IRF3) phosphorylation, which translocates into the nucleus after dimerization, inducing expression of type I IFN genes.^4^, ^36^ The production of IFNs is the prominent outcome of TLR3 and TLR4 pathways to counter viral infections, which in turn regulated by IRF3. Recently, it has been shown that phosphatidylinositol 5‐phosphate (PtdIns5P) can regulate IRF3 activation. This inositol lipid can bind to and facilitate complex formation between IRF3 and TBK1, leading to the IRF3 phosphorylation by TBK1, situated proximally.^37^ Furthermore, during viral infection, production of the inositol lipid, PtdIns5P, could be observed by evaluation of PIKfyve activity.^38^

## 3 ENDOGENOUS REGULATION OF TLR SIGNALING

Regulation of TLR signaling is achieved through various molecules that restrict it to an appropriate level to avoid any detrimental consequences in the form of autoimmune or inflammatory diseases. These regulatory molecules bind to key components of TLR signaling and quench their activities as reviewed elsewhere.^39^ The MyD88‐dependent pathway can be suppressed by spleen tyrosine kinase, Cbl‐b, and suppressor of cellular signaling 1 (SOCS1), while the TRIF arm is negatively regulated by sterile α‐ and armadillo‐motif‐containing protein (SARM) and TRAM adapter with Golgi dynamics domain (TAG).^40^, ^41^ The inhibition mechanisms of molecules can be unique or may overlap. Similarly, SOCS3 and deubiquitinating enzyme A (DUBA) negatively regulate TRAF3^42^ while A20, cylindromatosis, TANK, tripartite motif 38 (TRIM38), ubiquitin‐specific protease 4, and small heterodimer partner can negatively influence TRAF6 (Figure 1B).^39^, ^43^, ^44^ TAK1 activation is regulated by A20 and TRIM30α.^45^ NF‐κB is pivotal in TLR signaling; therefore, it is regulated by numerous molecules, including NF‐κB inhibitor δ (NFKBID), B‐cell lymphoma 3‐encoded protein (BCL‐3), activating transcription factor 3 (ATF3), Nurr1, and PDZ and LIM Domain 2 (PDLIM2).^46^ IRF3 is an important player in TRIF–dependent pathways that is suppressed by Pin1 and replication and transcription activator‐associated ubiquitin ligase (RAUL).^47^ Various microRNAs (miRNAs) have been implicated in mRNA level regulation of TLR signaling molecules, including miR‐21, ‐29, ‐126, ‐146a, ‐155, ‐199a, ‐148/152, and ‐466l.^39^ Moreover, cytokine mRNA stability can also be regulated by regulatory Regnase‐1 and tristetraprolin.^4^, ^48^ Collectively, TLR signaling homeostasis is established and maintained by these endogenous modulators (Figure 1B).

## 4 TLRS AND DISEASES

TLRs are involved in a wide spectrum of diseases that either directly or indirectly exacerbate the conditions. In recent years, many endeavors have been dedicated to delineate this relationship and compile data regarding the TLR involvement in various diseases.^4^, ^20^, ^49^, ^50^, ^51^ Here, we would like to present a brief overview of how TLRs influence the pathobiology of inflammatory, autoimmune, and cancerous diseases.^22^, ^23^, ^24^, ^52^

Sepsis is the worst outcome of host‐pathogen interaction and is the leading cause of death in United States.^53^, ^54^ The infection by Gram‐positive and Gram‐negative bacteria equally contribute to the development of sepsis where exaggerated immune response lead to multiorgan failure and septic shock.^55^ These bacteria harbor ligands that trigger TLR2 and TLR4; particularly, the presence of LPS significantly contributes into sepsis development. The septic shock is due to the body immune response rather than infection itself.^56^ For sepsis management, various TLR inhibitors are evaluated clinically, and new modalities are being devised recently.^57^

Chronic pulmonary obstructive disease (COPD) is characterized by the poor reversible air flow and bronchial inflammation.^52^, ^58^ This condition can also be worsened when TLRs react in response to viral infections. It has been observed that the COPD patients exhibit higher inflammatory cytokines, TNF‐α and CCL5 in infections.^59^ Among various treatments, the inhibition of TLRs can also be an approach to curb the COPD.^49^

The involvement of TLRs in RA, an inflammatory disease, is well known. The exact mechanism of RA initiation is yet debatable; however, it is believed that the PAMPs from commensal flora is crucial for RA initiation.^60^ After initial insult, an autocrine loop perpetuates that increases matrix metalloproteinases (MMP) and worsen the damage. Moreover, the DNA and peptidoglycan from intestinal bacteria have also been observed in RA joints.^61^ This result in damaged cells that will release DAMPs such as RNAs, HMGB1, S100‐A8; the presence of such molecules activate TLRs that over‐inflame the situation.

SLE is an autoimmune disease that featured autoantibodies against double‐stranded DNA and nucleic acid‐bound proteins that served both as diagnostic and prognostic markers; however, the initial events are still a mystery.^62^ SLE patients manifest deficiency in clearing apoptotic cells that promote the formation of the immune complex (IC), and these ICs can trigger the endosomal TLRs. The role of TLR7 (inflammatory) and TLR9 (protective) in SLE can be different due to variation in study samples among different studies; nevertheless, TLR9^−/−^ murine models displayed higher TLR7‐mediated inflammation concluding a regulatory role of TLR9.^63^, ^64^, ^65^

An autoimmune disease where the immune system destroys the fluid secreting glands, for instance, the salivary gland, has a potential TLR involvement and is known as Sjogren's syndrome (SS). The patients with SS exhibit higher TLR expression, with increased expression of inflammatory cytokines in response to TLR7 and TLR9 activation.^66^, ^67^

TLRs participation in cancers act as double‐edge swords; their activation can regress the tumor growth or conversely promote the tumor cells.^20^, ^67^ The accumulating data strongly advocate both aspects. Furthermore, it is now well‐acknowledged that the inflammation and cancer are strongly correlated in various diseases.^68^ Similarly, organs with higher PAMPs density such as gastrointestinal tract and skin are prone to TLR‐mediated oncogenesis along with the organs that expose to indirect TLR agonist such as the liver. The dual role of TLRs in cancers has a significant correlation with the length and amplitude of receptor activation. TLR4 has been reported to promote colon cancer, and its deficiency can alleviate the inflammation as well as tumor burden.^69^, ^70^ The liver cancer has also been related to TLR4 activation^71^; however, its role may be context dependent in skin cancer.^72^, ^73^ Similarly, TLRs are also critical for the cellular transformation in breast cancers, as reviewed before,^14^ can critically modulate the metabolism in the tumor microenvironment,^74^ and can regulate other signaling networks to favor pro‐ or anti‐tumor outcomes.^75^, ^76^, ^77^

## 5 TLR LIGANDS: ADJUVANTS VS DRUGS

TLR signaling activates innate immunity and assists in shaping adaptive immunity. Hence, TLR ligands are attractive for use in immunotherapy and are primarily exploited as adjuvants to specifically trigger humoral and/or cell‐mediated responses as reviewed elsewhere.^78^, ^79^ They can also magnify the immune response toward certain poorly antigenic targets. Therefore, in the majority of clinical trials, TLR ligands are evaluated as adjuvants.

The number of trials that involve TLR ligands as adjuvants (64%) are double than those considering TLR ligands as drugs (35%). This highlights the immune‐therapy role of TLRs in various diseases and their potential utilization for further exploration for immunomodulation therapy. Additionally, TLR activation can also alter other signaling pathways and it is desirable to cotarget multiple pathways with the aim of achieving improved treatment efficacy. Apart from many ongoing trials, Food and Drug Administration approved TLR ligands, MPLA^80^ (TLR4 agonist), and imiquimod^81^ (TLR7 agonist) could be highlighted to address adjuvant or drug roles. MPLA has been used in various vaccine formulations, for instance, Fendrix (Hepatitis B vaccine, GSK), as an adjuvant and imiquimod is famously used to cure viral diseases as a drug.^82^, ^83^ The majority of TLRs produce redundant responses (inflammatory vs antiviral); however, there are slight, but distinct, differences in outcomes.^84^ These differences are largely attributable to the relative roles of ligands and tissue‐dependent TLRs expression.^85^, ^86^

## 6 THERAPEUTIC INTERVENTIONS TARGETING TLRS

**Figure 2:**
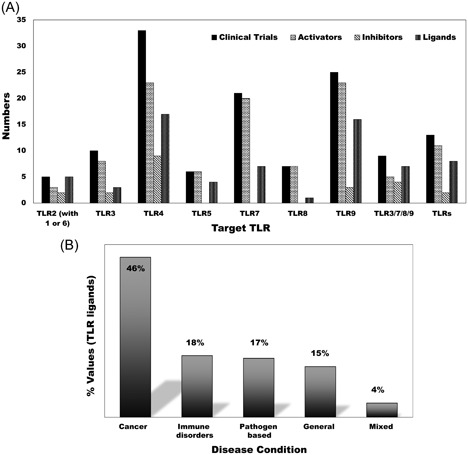
TLRs targeting ligands with respect to their relative clinical trials and disease conditions. A, The total number of clinical trials, activators (including agonists) and inhibitors (including antagonists), and the diversity of ligands are presented. The majority of ligands have been extensively pursued in different diseases, making it difficult to determine their exact numbers. The data indicate that total number of clinical trials exceeds the number of active drugs, suggesting the use of single drugs in multiple clinical trials. B, Clinical trial data showing the current status of drugs targeting TLRs from the disease perspectives. TLR ligands have been evaluated in multiple diseases including cancers, immune disorders, and viral and bacterial diseases. The largest proportion of clinical trials focuses on cancers, followed by immune disorders. “Mixed” indicates those cases where cancer and immune disease have been targeted simultaneously. The category “general” covers vaccination, clinical trials involving healthy volunteers, and those that are not covered by prior instances. This data was gathered from the clinical trials website (clinicaltrials.gov) using various keywords (cancers, immune disorders, TLR, TLRs, TLR1, TLR2, TLR3, TLR4, TLR5, TLR6, TLR7, TLR8, TLR9, and adjuvant) from June 2017 to Jan 2018. TLR, Toll‐like receptor

Given their vital roles in pathogen clearance, inflammation induction, and cancer pathogenesis, TLRs are attractive targets for manipulation of the immune system in favor of the patients. Therefore, many research centers and pharmaceutical companies are attempting to develop TLRs modulators (Figure 2A). Scaffolds of naturally occurring modulators are ideal candidates for targeting these receptors; thus, these have been heavily investigated in clinical studies and are emerging as a fruitful approach in clinical trials. An exhaustive search of the literature also supports this notion.

### 6.1 TLR2 (TLR1/TLR6)

TLR2, in combination with TLR1 or TLR6, detects the lipoproteins, diacyl lipid, or triacyl lipid, respectively, which makes it unique in forming functional heterodimers with other TLRs. Further, TLR2 interacts with modified proteins such as glyco‐ and lipoproteins, peptidoglycan, and zymosan, allowing it to detect a variety of PAMPs.^87^ This heterogeneity in TLR2 PAMP detection ranges across all types of pathogens, including viruses, bacteria, fungi, and parasites. The TLR2 expression has been detected in immune, endothelial, and epithelial cells,^88^ indicating that it is a functionally ubiquitous molecule. The homodimerization of TLR2 has been reported; however, further studies are required to confirm these findings.^8^, ^12^, ^89^ The ubiquitous nature and pivotal role of TLR2 make it an attractive drug‐target for various diseases; consequently, many clinical trials have been initiated to evaluate the efficacy of various lipopeptide derivates. Compounds being evaluated in clinical trials include lipopeptides, lipoproteins, oxidized low‐density lipoproteins, and TLR2 specific humanized IgG4 antibody, either alone or in various combinations (Table 2).

**Table 2:** TLR2 (with TLR1 or TLR6) targeting ligands in clinical trials

Ligand	Phase	Application	Target TLR adjuvant/drug	Sponsor/collaborators	NCT number	Type	Purpose
CBLB612	Phase 2	Breast cancer	TLR2 agonist/drug	Cleveland BioLabs, Inc	NCT02778763	Synthetic lipopeptide	Neutropenia/recover blood cells count
SV‐283 (NY‐ESO‐1)	Phase 1	Cancer	TLR2 agonist/adjuvant	SapVax LLC	NA	Combination of peptide and SM	Immune stimulation
ISA‐201	Phase 2	Head and neck tumor	TLR2/1 agonist/adjuvant	ISA Pharmaceuticals BV	NCT02821494	Combination of peptide and SM, peptide	DC maturation
OPN‐305‐110	Phases 1 and 2	Second‐line and first line lower risk myelodysplastic syndrome	TLR2 antagonist/drug	Opsona Therapeutics Ltd	NCT03337451	Monoclonal antibody	Anti‐inflammation
OPN‐305	Phase 2	Myelodysplastic syndrome, inflammatory disease, pancreas tumor, kidney transplant rejection	TLR2 antagonist/drug	Opsona Therapeutics Ltd	NCT02363491	Monoclonal antibody	Anti‐inflammation

Abbreviations: DC, dendritic cells; NA, not available; NCT, national clinical trial; SM, small molecule; TLR, Toll‐like receptor.

The most recent ligands, such as CBLB612 (synthetic lipopeptide TLR2 agonist), ISA‐201 (peptide agonist for TLR2), OPN‐305 (TLR2 antagonizing IgG4 monoclonal antibody), are in phase 2 in clinical trials primarily for oncogenic therapy, and act both as drugs and as adjuvants (Figure 2B).^90^, ^91^ The chemical constituents of these molecules are either lipoprotein or protein derivates, indicating that TLR2 can be targeted using mimetics of its natural ligands. This is not an absolute requirement; however, it is useful to note the existing therapeutic trend while targeting TLR2. Moreover, other than OPN‐305, the majority of molecules in phase 2 trials are agonists of TLR2, highlighting the importance of TLR2 activation in the context of malignancies. Small molecule‐based therapeutics have potential side effects that can be overcome by the application of biologics, including monoclonal antibodies (OPN‐305).^90^ The inhibition of TLR2 overactivation using OPN‐305 has potential applications in the treatment of inflammatory diseases.

**Figure 3:**
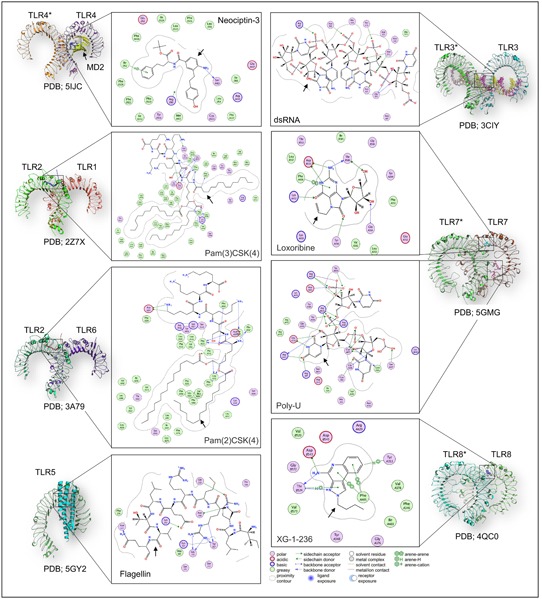
TLRs with bound ligands. The ligand binding mechanism of the extracellular TLRs (left, TLR1, 2, 4, 5, 6) and endosomal TLRs (right, TLR3, 7, 8) has been presented. Each monomer has been labeled; however, for the homodimers, the other monomer has been labeled with asterisk (*). In the case of TLR5, flagellin‐bound single monomer has been given. The respective protein databank (PDB) ID has also been given at the bottom of each structure. TLRs recognize several molecules, including protein, lipopeptide, small molecules and nucleic acids, and the bound ligand with individual TLR has been shown in 2D interaction diagram. The color code for the 2D interaction is given at the bottom of the figure. Black arrows indicate the bound TLR‐ligand. dsRNA, double‐stranded RNA; TLR, Toll‐like receptor; 2D, two‐dimensional [Color figure can be viewed at wileyonlinelibrary.com]

TLR2 in TLR2/1 or TLR2/6 complexes exhibits a cavity on the binding junction of its convex side that allows the docking of Pam3CSK4 and other TLR2‐modulating ligands.^12^, ^92^ Pam3CSK4 has two esters and one amide bound lipid chains. The ester chains interact with TLR2, while the amide bound lipid chain can be accommodated into the hydrophobic cavity provided by TLR1 (Figure 3).^12^, ^93^ The TLR2/1 complex can further be stabilized by interprotein hydrogen bonding and hydrophobic interactions.^12^ The hydrophobic cavity in TLR1 has been mutated with bulky amino acids (Met338 and Leu360 to Phe338/360) in TLR6 to make binding of any lipid chain unfavorable, explaining the diacyl requirement for TLR2/6 complex formation.

### 6.2 TLR3

TLR3 forms homodimer and signals in an exclusively TRIF–dependent manner in response to viral infections (double‐stranded RNA [dsRNA]) and stimulates the production of IFNs. The only known agonist for this TLR is poly‐ICLC (and its derivatives), which is being investigated in various clinical trials.^94^, ^95^ The success and ubiquitous nature of poly‐ICLC led to the belief that this was the only realistic possibility for targeting this TLR; however, recent studies have identified other small molecules that can either inhibit or activate TLR3.^96^, ^97^ These alternatives will not be available for clinical trials for a considerable period of time. There are a few clinical trials that involve anti‐TLR3 antibody to evaluate its efficacy in healthy individuals and asthmatic patients.^98^ The success of these proof‐of‐concept studies will lay the foundation of antibody‐based endosomal TLRs targeting in various diseases. However, in rhinoviral infection, the antibody could not demonstrate any improvement in asthmatic condition.^99^ Targeting of TLR3 is currently used as adjuvant therapy, along with other drugs or vaccines, against a variety of cancers; nonetheless, the sole activation of TLR3 to curb any disease has yet to be successfully explored (Table 3).

**Table 3:** TLR3 targeting ligands in clinical trials

Ligand	Phase	Application	Target TLR adjuvant/drug	Sponsor/collaborators	NCT number	Type	Purpose
Poly‐ICLC	Phases 1 and 2	Low‐grade B‐cell lymphoma	TLR3 agonist/adjuvant	Joshua Brody, Icahn School of Medicine	NCT01976585	Synthetic dsRNA	DC activation
Poly‐ICLC (NY‐ESO‐1)	Phases 1 and 2	Melanoma	TLR3 agonist/adjuvant	Nina Bhardwaj, Ludwig Institute for Cancer Research, Oncovir Inc, CRI New York City, Icahn School of Medicine	NCT01079741	Synthetic dsRNA	Activating humoral and T cell immunity
Poly‐ICLC	Phases 1 and 2	Head and neck squamous cell carcinoma, sarcoma, Merkel cell carcinoma, cutaneous T‐cell lymphoma, melanoma, renal‐ bladder‐breast and prostate cancer	TLR3 agonist/adjuvant	Ludwig Institute for Cancer Research, MedImmune LLC, CRI New York City	NCT02643303	Synthetic dsRNA	Tumor microenvironment modulation
Poly‐ICLC (Hiltonol)	Unknown	Primary ovarian cancer, fallopian tube cancer, primary peritoneal cancer	TLR3 agonist/adjuvant	Abramson Cancer Center of the University of Pennsylvania	NCT02452775	Synthetic dsRNA, carboxymethylcellulose, poly‐l‐lysine	Immune stimulation
Poly‐ICLC (Romidepsin)	Phase 1	Cutaneous T‐cell lymphoma	TLR3 agonist/adjuvant	New York University School of Medicine, Ludwig Institute for Cancer Research	NCT02061449	Synthetic dsRNA	Immune stimulation
Poly‐ICLC (Pembrolizumab)	Phases 1 and 2	Metastatic colon cancer, solid tumor	TLR3 agonist/adjuvant	Samir N. Khleif, Oncovir Inc, Merck Sharp and Dohme Corp, Augusta University	NCT02834052	Synthetic dsRNA	Immune stimulation
Poly‐ICLC	Phases 1 and 2	Chronic HIV‐1 infection	TLR3 agonist/adjuvant	The Campbell Foundation; Oncovir, Inc, National Institutes of Health (NIH), National Institute of Allergy & Infectious Diseases (NIAID), Nina Bhardwaj, Icahn School of Medicine at Mount Sinai	NCT02071095	Synthetic dsRNA	Immune stimulation
Poly‐ICLC (DCVax‐001)	Phase 1	HIV‐1 infection	TLR3 agonist/adjuvant	Rockefeller University	NCT01127464	Synthetic dsRNA	Immune stimulation
PRV‐300	Phase 1	Healthy	TLR3 antagonist/drug	Janssen Research & Development, LLC	NCT02008279	Anti‐TLR3 antibody	Anti‐inflammatory
PRV‐300	Phases 1 and 2	Asthma	TLR3 antagonist/drug	Centocor, Inc	NCT01195207	Anti‐TLR3 antibody	Anti‐inflammatory

Abbreviations: DC, dendritic cells; dsRNA, double‐stranded RNA; HIV, human immunodeficiency virus; NCT, national clinical trial; TLR, Toll‐like receptor.

Poly‐ICLC is a synthetic complex of polyinosinic‐polycytidylic acid (nucleic acid mimetics and pathway intermediates), carboxymethylcellulose, and poly‐l‐lysine (stabilizers). As dsRNA is a natural ligand with relatively low stability, its mimetics could be an affordable means of activating this TLR. Activation of TLR3 depends on dsRNA binding at two opposite sides of its ectodomain, which favorably relocates the C‐terminus of the ECD to facilitate further interactions and increased stabilization.^100^, ^101^ TLR3 interacts with the nucleotide backbone, rather than nucleotide bases, which explains its activation via multiple nucleotide combinations (Figure 3).^100^

### 6.3 TLR4

TLR4 is the only TLR that can function both at the cell membrane and in the endosome, and that can signal through MyD88‐ and TRIF–dependent pathways.^102^, ^103^ This has led to the evolution of additional precautions, such as an extensive ligand detection mechanism (cluster of differentiation 14, lipid binding protein, and coreceptor myeloid differentiation factor 2 [MD2]) and signal propagation mechanism (requirement of MAL for MyD88 and TRIF‐related adapter molecule [TRAM] for TRIF signaling pathways).

Among TLRs, only TLR4 has a suitable ligand binding pocket provided by MD2, rather than by the ectodomain of TLR4. This also provides an additional means of TLR4 (in)activation,^104^ since MD2 has a large hydrophobic cavity and lipid A derivatives are suitable binding molecules; however, other methods, such as disruption of MD2 binding with TLR4 or inhibition of interaction with the activating ligand, have also been explored.^105^ In case of MD2 and lipid A interaction, the lipid with six acyl chains (lipid VI‐A) can fully occupy the pocket and reorient the side chain of F126 amino acid into the binding cavity. This creates a favorable environment for the other TLR4/MD2 to dock properly (Figure 3).^106^ However, in case of a lower number of acyl chains, their binding is unable to reorient the side chain that, in turns, creates a steric hindrance for other heterodimer, leading to TLR4 inhibition.^11^, ^104^, ^107^

Prominent ligands that activate or inhibit TLR4 include lipid VI‐A and its derivatives (lipid 4A [antagonist], monophosphoryl lipid A [MPLA, a weak agonist],^108^ and glucopyranosyl lipid adjuvant [GLA, an agonist]),^109^ and recent studies have also evaluated antibody or peptide‐based drugs.^105^, ^110^ Among the TLRs, TLR4 has been extensively evaluated in the highest number of clinical trials and is of interest as a target for treatment of a variety of pathologies including cancers, viral infection, immune diseases, and inflammation. Both the agonistic and antagonistic aspects of TLR4 signaling pathways are being explored (Table 4). In addition to inhibition of MD2‐mediated TLR4 signaling, the interaction of HMGB1 with TLR4 has also been considered in recent trials to improve the efficacy of anticancer drugs (https://clinicaltrials.gov/ct2/show/NCT02995655). Besides direct modulation, the addition of a constitutively active form of TLR4 as a vaccine substitute (https://clinicaltrials.gov/ct2/show/NCT02888756) and inhibition of dipeptidyl peptidase‐4 (DPP4) that induces IL‐6 expression through TLR4 are also the subjects of therapeutic evaluations.^111^

**Table 4:** TLR4 targeting ligands in clinical trials

Ligand	Phase	Applications	Target TLR adjuvant/drug	Sponsor/collaborators	NCT number	Type	Purpose
GLA‐SE (MART‐1 antigen)	Phase 1	Stage IIA–IV skin melanoma	TLR4 agonist/adjuvant	Mayo Clinic, NCI	NCT02320305	Glycolipid	Immune stimulation
LPS	Phase 1	Asthma	TLR4 agonist/adjuvant	John Sundy, Duke University	NCT00671892	Glycolipid	Prospective inflammation
GLA‐SE	Phase 1	Stage III/IV adult soft tissue sarcoma	TLR4 agonist/adjuvant	Fred Hutchinson Cancer Research Center, NCI	NCT02180698	Glycolipid	Immune stimulation
GSK1795091	Phase 1	Cancer	TLR4 agonist/drug	GSK	NCT02798978	Glycolipid	Immune stimulation
G100	Phases 1 and 2	Follicular lymphoma (marginal zone allowed during dose escalation only)	TLR4 agonist/adjuvant	Immune Design, Merck Sharp & Dohme Corp	NCT02501473	Glycolipid	Tumor microenvironment alteration, DC, T and other immune cells activation
GLA‐AF, GLA‐SE	Phase 1	Healthy volunteers	TLR4 agonist/adjuvant	Rockefeller University, IDRI Corporation, Immune Design	NCT01397604	Glycolipid	Immune stimulation, DC activation
CCRE	Phase 1	aHealthy individual	TLR4 agonist/adjuvant	University of North Carolina, Chapel Hill, Environmental Protection Agency (EPA)	NCT02847247	LPS	Inflammation
Endotoxin	Phase 1	Endotoxemia	TLR4 agonist/adjuvant	Radboud University	NCT00184990	SM	Inflammation
GLA	Phase 1	Hookworm infection	TLR4 agonist/adjuvant	Baylor College of Medicine, George Washington University	NCT01717950	Glycolipid	Immune stimulation
Eritoran	Phase 2	Insulin sensitivity	TLR4 antagonist/drug	The University of Texas Health Science Center at San Antonio	NCT02321111	Glycolipid	Lipid‐induced insulin resistance
NI‐0101	Phase 2	Rheumatoid arthritis	TLR4 inhibitor/drug	NovImmune SA	NCT03241108	Anti‐TLR4 antibody	TLR4 blocker, anti‐inflammatory
NI‐0101	Phase 1	Healthy volunteers	TLR4 inhibitor/drug	NovImmune SA	NCT01808469	Anti‐TLR4 antibody	TLR4 blocker, anti‐inflammatory
CX‐01	Phase 1	Refractory Myelodysplastic Syndrome and Acute Myeloid Leukemia	TLR4 inhibitor/adjuvant	Washington University School of Medicine, Cantex Pharmaceuticals	NCT02995655	Polysaccharide	Bone marrow microenvironment disruptor
Eritoran	Phase 2	Insulin sensitivity	TLR4 antagonist/drug	Nicolas Musi, The University of Texas Health Science Center at San Antonio	NCT02267317	Glycolipid	Anti‐inflammatory
MPL (Grass MATA)	Phase 3	Type 1 hypersensitivity	TLR4 agonist/adjuvant	Allergy Therapeutics Plc	NCT00414141	Glycolipid	Anti‐inflammatory
MPL (Grass MATA)	Phase 2	Allergy	TLR4 agonist/adjuvant	Allergy Therapeutics Plc	NCT02582073	Glycolipid	Anti‐inflammatory
GLA‐SE	Phase 1	Merkel cell carcinoma	TLR4 agonist/adjuvant	Immune Design Corp	NCT02035657	Glycolipid	Immune stimulation
GLA (H5 VLP vaccine)	Phase 2	Influenza virus infection	TLR4 agonist/adjuvant	Medicago Inc	NCT01991561	Combination of protein and SM	Immune stimulation
PEPA‐10	Phase 2	Cancer	TLR4 agonist/adjuvant	Immunovo BV	NA	SM	Immune stimulation
PET‐lipid A (ONT‐10)	Phase 1	Advanced Breast and Ovarian Carcinoma	TLR4 agonist/adjuvant	Cascadian Therapeutics Inc	NCT02270372	Glycolipid	Immune stimulation
PET‐lipid A (ONT‐10)	Phase 1	Solid tumors	TLR4 agonist/adjuvant	Cascadian Therapeutics Inc	NCT01556789	Glycolipid	Immune stimulation
PET‐lipid A (ONT‐10)	Phase 1	Solid tumors	TLR4 agonist/adjuvant	Cascadian Therapeutics Inc	NCT01978964	Glycolipid	Immune stimulation
GLA (H7N9 influenza vaccine)	Phase 1	Influenza virus A infection	TLR4 agonist/adjuvant	Medicago Inc	NCT02022163	Glycolipid	Enhance seroprotective antibody titers
G‐305	Phase 1	Cancer	TLR4 agonist/adjuvant	Immune Design Corp	NCT02015416	Combination of protein and SM	Immune stimulation
Lipid A (SAR‐439794)	Phase 1	Peanut hypersensitivity	TLR4 agonist/adjuvant	Sanofi, Immune Design Corp	NCT03463135	Glycolipid	Immune stimulation
GLA	Phase 1	Schistosomiasis	TLR4 agonist/adjuvant	Immune Design Corp	NCT02337855	Glycolipid	Immune stimulation
JKB‐121	Phase 2	Nonalcoholic steatohepatitis	TLR4 antagonist/drug	TaiwanJ Pharmaceuticals	NCT02442687	SM	Anti‐inflammatory
GLA‐SE (CMB‐305)	Phase 2	Sarcoma and its various forms	TLR4 agonist/adjuvant	Immune Design Corp	NCT02609984	Glycolipid	Immune stimulation, DC activation
GLA‐SE (CMB‐305)	Phase 2	Sarcoma, melanoma, nonsmall‐lung cancer	TLR4 agonist/adjuvant	Immune Design Corp	NCT02387125	Glycolipid	Immune stimulation, DC activation
JKB‐122	Phase 2	Chronic hepatitis C	TLR4 antagonist/drug	TaiwanJ Pharmaceuticals	NCT02293941	SM	Anti‐inflammatory
JKB‐122	Phase 2	Autoimmune hepatitis, nonalcoholic fatty liver disease	TLR4 antagonist/drug	TaiwanJ Pharmaceuticals	NCT02556372	SM	Anti‐inflammatory
Ibudilast (aka MN‐166)	Phase 2	Alcoholism, neuropathic pain, amphetamine and opiate dependence, glioblastoma, traumatic brain injury	TLR4 antagonist/drug	MediciNova Inc	NCT01860807	SM	Proinflammatory cytokine suppression
Intralipid (20%)	NA	Diabetes, obesity	TLR4 expression	The University of Texas Health Science Center at San Antonio, NIDDK	NCT01740817	Lipid, SM	TLR4 expression

Abbreviations: CCRE, clinical center reference endotoxin; GLA, glycopyranosyl lipid A; GSK, GlaxoSmithKline; NA, not applicable; NCI, National Cancer Institute; NCT, national clinical trial; NIDDK, National Institute of Diabetes and Digestive and Kidney Diseases; SM, small molecule; TLR, Toll‐like receptor.

### 6.4 TLR5

TLR5 detects the bacterial monomeric flagella and mounts an immune response.^112^, ^113^ It triggers the MyD88‐dependent pathway in response to enterobacterial invasion and maintains intestinal homeostasis. TLR5 is expressed in almost all cell types with prominent expression in mucosal dendritic cells (DC); however, literature discussing TLR5 is scarce.^114^, ^115^ From a therapeutic perspective, all clinical trials targeting TLR5 include use of recombinant flagellin protein.^116^, ^117^, ^118^, ^119^ Additionally, small molecules to block the TLR5‐flagellin interaction are being tested in preclinical studies.^120^ In the majority of therapeutic settings, ligands for TLR5 act as adjuvants rather than as stand‐alone drugs, enhancing the efficacy and potency of vaccine candidates (Table 5). TLR5 can be an attractive target because it detects only protein. Short peptides derived from flagellin can be used as activators, while modified forms of such peptides can inhibit TLR5.^118^, ^121^ Recently, the crystal structure of zebrafish TLR5 with flagellin was determined, providing insights into its mode of activation (Figure 3).^122^ The leucine‐rich repeat 9 (LRR9) region in TLR5 has a critical role, and Arg89, Glu114, and Leu93 from flagellin form a hotspot with chemical and geometric complementarity.^122^ These regions on both proteins should be explored further to design novel therapeutics.

**Table 5:** TLR5 targeting ligands in clinical trials

Ligand	Phase	Application	Target TLR adjuvant/drug	Sponsor/collaborators	NCT number	Type	Purpose
Mobilan (M‐VM3)	Phases 1 and 2	Prostate cancer	TLR5 agonist/adjuvant	Panacela Labs LLC	NCT02844699	RP	Immune stimuation
Mobilan (M‐VM3)	Phase 1	Prostate cancer	TLR5 agonist/adjuvant	Panacela Labs LLC	NCT02654938	RP	Immune stimulation
Entolimod (aka CBLB502)	Phase 1	Unspecified adult solid tumor	TLR5 agonist/adjuvant	Roswell Park Cancer Institute, NCI, Cleveland BioLabs Inc	NCT01527136	RP	Immune stimulation
VAX125	Phase 2	Influenza	TLR5 agonist/adjuvant	VaxInnate Corporation	NCT00966238	RP	Immune stimulation
VAX102 (flagellin.HuM2e)	Phase 1	Influenza	TLR5 agonist/adjuvant	VaxInnate Corporation; Bill & Melinda Gates Foundation	NCT00603811	RP	Immune stimulation
Entolimod (radiation therapy)	Phase 1	Mucositis, various types of squamous cell carcinoma of various tissues	TLR5 agonist/adjuvant	Roswell Park Cancer Institute, NCI, Cleveland BioLabs Inc	NCT01728480	RP	Immune stimulation

Abbreviations: NCI, National Institute of Cancer; NCT, national clinical trial; RP, recombinant protein; TLR, Toll‐like receptor.

### 6.5 TLR7/8

TLR7 and TLR8 are functionally active in the endosomal compartment, use MyD88 adapter molecules and are activated by single‐stranded RNA (ssRNA).^123^, ^124^ Majority of ligands in clinical trials that target TLR7/8 are small molecules (eg, imiquimod [R837], resiquimod, or GSK2245035), and most are derivatives of imidazoquinoline, a tricyclic organic molecule.^125^, ^126^, ^127^ (Tables 6, 7, 8). There are functional differences between these two TLRs; for example, plasmacytoid DC and monocytes can be directly activated by TLR7 agonists; however, other than monocytes, TLR8 agonists can directly activate mDCs and monocyte‐derived DCs. TLR7 agonists were more potent when compared with TLR8 agonists regarding antiviral responses in the form of IFN, I‐TAC (IFN‐inducible T‐cell α chemoattractant), and IFN‐regulated cytokines from human peripheral blood mononuclear cells (PBMC).^128^ Proinflammatory responses, such as expression of IL‐12, TNF‐α, and macrophage inflammatory proteins‐1α (MIP‐1α) were enhanced by TLR8 agonism when compared with TLR7, leading to characteristic differential cell induction profiles.

**Table 6:** TLR7 targeting ligands in clinical trials

Ligand	Phase	Application	Target TLR adjuvant/drug	Sponsor/collaborators	NCT number	Type	Purpose/mechanism
GSK2245035	Phase 2	Asthma and rhinitis	TLR7 agonist/drug	GSK, PATH	NCT01607372	SM	Type 1 IFN induction
Imiquimod (R837)	Phases 1 and 2	Metastatic and recurrent breast cancer	TLR7 agonist/adjuvant	New York University School of Medicine, NCI	NCT01421017	SM	Adaptive immune stimulator
Imiquimod	Phase 1	Melanoma and metastatic cancer	TLR7 agonist/drug	University of Oklahoma, NCI	NCT00453050	SM	Immune stimulation
Imiquimod	Phase 2	Breast cancer and breast neoplasms	TLR7 agonist/drug	New York University School of Medicine	NCT00899574	SM	Immune stimulation
852A	Phase 2	Breast, ovarian, endometrial, and cervical cancer	TLR7 agonist/drug	Masonic Cancer Center University of Minnesota, Pfizer	NCT00319748	SM	DC activation, IFN‐α secretion
Imiquimod	Phase 3	Influenza viral infection	TLR7 agonist/adjuvant	The University of Hong Kong	NCT02103023	SM	Improve vaccine immunogenicity against influenza virus
Imiquimod	Phase 2	HPV	TLR7 agonist/drug	Medical University of Vienna	NCT00941811	SM	Immune stimulation
Imiquimod (intradermal HBVv)	Phases 2 and 3	Renal failure	TLR7 agonist/adjuvant	The University of Hong Kong	NCT02621112	SM	Improving vaccine immunogenicity
Imiquimod (influenza vaccine)	Unknown	Chronic illness	TLR7 agonist/adjuvant	The University of Hong Kong	NCT01508884	SM	Activation of APC
GS‐9620	Phase 2	Chronic hepatitis B	TLR7 agonist/adjuvant	Gilead Sciences	NCT02166047	SM	Activation of pDC
Single or multiple GS‐9620	Phase 1	Hepatitis B	TLR7 agonist/adjuvant	Gilead Sciences	NCT01590654	SM	Activation of pDC, Immune stimulation
Imiquimod	Unknown	Photoaged skin and normal skin	TLR7 agonist/adjuvant	University of Michigan	NCT02889159	SM	Immune stimulation
RO6864018 (aka ANA773, RO‐6864018)	Phase 1	Healthy volunteer	TLR7 agonist/drug	Hoffmann‐La Roche	NCT02015715	SM	Immunomodulator, Immune stimulation
RO7020531	Phase 1	Chronic Hepatitis B	TLR7 agonist/drug	Hoffmann‐La Roche	NCT02956850	SM	Immune stimulation, B and T cell activations
RO6864018	Phase 2	Chronic Hepatitis B	TLR7 agonist/drug	Hoffmann‐La Roche	NCT02391805	SM	Immunomodulator, Immune stimulation
GSK2245035	Phase 2	Mild asthma and allergic rhinitis	TLR7 agonist/drug	GlaxoSmithKline	NCT01788813	SM	Immune stimulation
GSK2445053	Phase 1	Rhinitis, allergic	TLR7 agonist/drug	GlaxoSmithKline	NCT01480271	SM	Induction of IFNα
GSK2245035	Phase 2	Allergy rhinitis, asthma, and respiratory tract allergy	TLR7 agonist/drug	GlaxoSmithKline	NCT02833974	SM	Immune stimulation
Imiquimod	Phase 3	Actinic keratosis	TLR7 agonist/drug	Graceway Pharmaceuticals, LLC	NCT00894647	SM	Immune stimulation
Imiquimod	Phase 4	Actinic keratosis	TLR7 agonist/drug	MEDA Pharma GmbH & Co. KG	NCT00777127	SM	Immune stimulation
Imiquimod	Approved (China, Dec 2004)	Keratosis, mycosis fungoides, verruca vulgaris, condyloma, basal cell carcinoma, and molluscum contagiosum infection	TLR7 agonist/adjuvant	Mochida Pharmaceutical Co Ltd, 3 M Pharmaceuticals, Valeant Pharmaceuticals International Inc, iNova Pharmaceuticals Pty Ltd, Intendis GmbH, Meda AB	NCT01453179	SM	Immune stimulation

Abbreviations: GSK, GlaxoSmithKline; HPV, human papilloma virus; IFN, interferon; NCI, National Cancer Institute; NCT, national clinical trial; SM, small molecule; TLR, Toll‐like receptor.

**Table 7:** TLR3/7/8/9 targeting ligands in clinical trials

Ligand	Phase	Application	Target TLR adjuvant/drug	Sponsor/collaborators	NCT number	Type	Purpose/mechanism
Resiquimod, poly‐ICLC	Phase 2	Glioma, anaplastic astrocytoma, anaplastic astro‐oligodendroglioma	TLR3/7/8 agonist/adjuvant	Jonsson Comprehensive Cancer Center	NCT01204684	SM, synthetic dsRNA	Antitumor and antiviral immune stimulation
Poly‐ICLC, resiquimod (R848)	Phases 1 and 2	Melanoma and its metastatic mucosal variants	TLR3/7/8 agonist/adjuvant	Craig L Slingluff, Jr, University of Virginia	NCT02126579	SM, synthetic dsRNA	Antitumor immune stimulation
Resiquimod (R848)	Phase 1	Influenza vaccination in seniors	TLR7/8 agonist/adjuvant	University of British Columbia	NCT01737580	SM	Immune stimulation
MEDI9197 (durvalumab)	Phase 1	Solid tumors, cutaneous T cell lymphoma	TLR7/8 agonist/adjuvant	MedImmune LLC	NCT02556463	SM	Improving antigen presentation
Resiquimod (NY‐ESO‐1)	Phase 1	Tumors	TLR7/8 agonist/adjuvant	Nina Bhardwaj, CRI New York City, Icahn School of Medicine at Mount Sinai	NCT00821652	SM	Immune stimulation
IMO‐8400	Phases 1 and 2	Waldenstrom's macroglobulinemia	TLR7/8/9 antagonist/adjuvant	Idera Pharmaceuticals, Inc	NCT02092909	Oligonucleotide antagonist	Immune suppression, TLR7/8/9 signaling blocker
IMO‐8400	Phase 2	Plaque psoriasis	TLR7/8/9 antagonist/drug	Idera Pharmaceuticals, Inc	NCT01899729	Oligonucleotide antagonist	Immune suppression
CPG‐52364	Phase 1	Healthy volunteers	TLR7/8/9 antagonist/drug	Pfizer	NCT00547014	SM	Immune suppression
IMO‐8400	Phases 1 and 2	Diffuse large B cell lymphoma	TLR7/8/9/ antagonist/drug	Idera Pharmaceuticals, Inc	NCT02252146	Oligonucleotide antagonist	Immune suppression

Abbreviations: CRI, Cancer Research Institute; dsRNA, double‐stranded RNA; NA, not available; NCT, national clinical trial; SLE, systematic lupus erythematosus; SM, small molecule; TLR, Toll‐like receptor.

**Table 8:** TLR8 targeting ligands in clinical trials

Ligand	Phase	Application	Target TLR adjuvant/drug	Sponsor/collaborators	NCT number	Type	Purpose/mechanism
VTX‐2337 (Motolimod)	Phase 1	A variety and different stages of metastatic squamous neck cancer with occult primary squamous cell carcinoma	TLR8 agonist/adjuvant	University of Washington, NCI	NCT01334177	SM	Immune stimulation
VTX‐2337	Phase 1	For various types and stages of colorectal, pancreatic, breast, melanoma, non‐small cell lung carcinoma, pancreatic, renal cell carcinoma and solid neoplasm	TLR8 agonist/adjuvant	Mayo Clinic, NCI	NCT02650635	SM	Immune stimulation
VTX‐2337	Phase 1	Various types of ovarian cancers and fallopian tube carcinoma, recurrent ovarian carcinoma	TLR8 agonist/adjuvant	Gynecologic Oncology Group, NCI	NCT01294293	SM	Immune stimulation
VTX‐2337	Phase 2	Epithelial ovarian cancer, fallopian tube cancer, primary peritoneal cancer	TLR8 agonist/adjuvant	VentiRx Pharmaceuticals Inc, Gynecologic Oncology Group	NCT01666444	SM	Immune stimulation
VTX‐2337 (with radiotherapy)	Phases 1 and 2	Low grade B‐Cell lymphoma	TLR8 agonist/adjuvant	VentiRx Pharmaceuticals Inc, Stanford University	NCT01289210	SM	Immune stimulation
VTX‐2337	Phase 2	Carcinoma, squamous cell of head and neck	TLR8 agonist/adjuvant	VentiRx Pharmaceuticals Inc	NCT01836029	SM	Immune stimulation
VTX‐2337 (with anti‐PD‐L1 antibody MEDI4736)	Phases 1 and 2	Ovarian cancer	TLR8 agonist/adjuvant	Ludwig Institute for Cancer Research, MedImmune LLC, VentiRx Pharmaceuticals Inc, CRI New York City	NCT02431559	SM	Immune stimulation

Abbreviations: CRI, Cancer Research Institute; NCI, National Cancer Institute; NCT, national clinical trial; PD‐L1, programmed death‐ligand 1; SM, small molecule; TLR, Toll‐like recptor.

TLR7 agonists have been actively studied in phase 1 and 2 trials aiming to curb the persistent viral load in HIV‐ and HBV‐infected individuals.^129^ Moreover, various prodrug (pharmacologically active after metabolism) approaches have also targeted TLR7 (RO6870868 [single prodrug] or RO6864018 [double prodrug]), and use of these as TLR7 agonists was useful in treating hepatitis B infection in phase 1 clinical trials. The results of this trial were promising and paved the way for phase 2 trials (https://clinicaltrials.gov/ct2/show/NCT02015715).

TLR8 can also be activated by ssRNA as natural ligand and by VTX‐2337 (motolimod), a synthetic small molecule selective for TLR8 and is being evaluated in clinical trials.^124^, ^130^ TLR8 is a less studied receptor, as its roles overlap with those of TLR7, with which it shares multiple features. When treated with VTX‐2337, TLR8 stimulates TNF‐α and IL‐12 production at lower concentrations in human PBMCs. It also induces TNF‐α and IL‐12 secretion from monocytes and myeloid DCs through the NF‐κB pathway. IFNγ secretion was observed when NK cells were treated with VTX‐2337, which can enhance the lytic capability and antibody‐dependent cell‐mediated cytotoxicity of NK cells.^130^ VTX‐2337 also improves the efficacy of pegylated liposomal doxorubicin in treatment of ovarian cancer in a mouse model with humanized immune system that has been reconstituted with human CD34^+^ cells.^131^ This is the only ligand molecule that has been actively evaluated for treatment of a variety of cancers, including head and neck cancer, colorectal, pancreatic, melanoma, breast, renal cell carcinoma, nonsmall–cell lung carcinoma, and other solid neoplasms. In the majority of cases, VTX‐2337 was used in combination with other drugs; however, it is also being evaluated as a stand‐alone drug for treatment of lymphoma.^130^

TLR7 and TLR8 share similar activation patterns, both have z‐loops involved in ssRNA recognition, and both possess two binding sites; the first binding site binds guanosine and uridine in TLR7 and TLR8, respectively, while the second binds ssRNA in both cases (Figure 3).^132^ In TLR7 ssRNA binding primes the receptor for guanosine binding and subsequent dimerization, while synthetic molecules, such as R848, can activate TLR7 without the need for ssRNA.^133^, ^134^ Importantly, TLR7 remains monomeric in the absence of any ligand and dimerizes in response to ligand binding; however, its dimer conformation is similar to TLR8 and TLR9. TLR8, on the other hand, is a naturally occurring weak dimer that undergoes conformational change upon ligand binding. The Z‐loop may have an important regulatory role; when it is cleaved from TLR8, TLR8 forms a tight dimer and initiates signaling in the absence of ligand.^135^

### 6.6 TLR9

TLR9 senses CpG DNA in endosomes and induces the IFN response.^136^, ^137^ TLR9 is involved in numerous diseases and has been targeted by various therapeutic approaches. All of the ligands tested in clinical trials that exclusively target TLR9 are either nucleotides or nucleotide derivatives. There are various types of CpG DNAs that are being evaluated in different trials for treatment of diverse conditions. AZD1419 is a C‐type CpG‐based inhaled TLR9 agonist for treatment of asthma and to stimulate IFNs production in lungs. This treatment was classified as well‐tolerated and safe in phase 1 human trials with potential disease‐modifying characteristics and is a promising new therapeutic for use in various immune diseases.^138^ CYT003 was initially found to be effective; however, its effects were not confirmed in phase 2 clinical trials where 35 patients were treated with varying doses of CYT003.^139^ Another TLR9 agonist, EMD 1201081, was evaluated in phase 2, open‐label, randomized trial in patients with head and neck cancer, and was found to be ineffective in the tested dose regimen.^140^ GNKG168 is another CpG‐based molecule that can induce CD8^+^ T cell antitumor cytotoxic responses; however, it was withdrawn in clinical phase 1 because of sponsor reluctance to further support the study^141^ (NCT01743807) (Tables 7 and 9).

**Table 9:** TLR9 targeting ligands in clinical trials

Ligand	Phase	Application	Target TLR adjuvant/drug	Sponsor/Collaborators	NCT Number	Type	Purpose/ Mechanism
MGN1703 (Ipilimumab)	Phase 1	Melanoma	TLR9 agonist/adjuvant	MD Anderson Cancer Center, Mologen AG	NCT02668770	DNA‐based molecule	Antitumor immune stimulation
SD‐101	Phases 1 and 2	Lymphoma and its various forms	TLR9 agonist/adjuvant	Robert Lowsky, NCI, Stanford University	NCT02254772	CpG‐C class oligodeoxynucleotide	Antitumor immune stimulation
SD‐101	Phases 1 and 2	Grade 1/2/3 follicular lymphoma and recurrent and refractory follicular lymphoma	TLR9 agonist/drug	Robert Lowsky, NCI, Stanford University	NCT02927964	CpG‐C class oligodeoxynucleotide	Antitumor immune stimulation
CpG vaccine (autologous tumor cell)	Phase 1	Colorectal neoplasms, anal, colon, and rectal cancers	TLR9 agonist/adjuvant	Stanford University	NCT00780988	Oligonucleotide	Antitumor immune stimulation
CYT003	Phase 2	Moderate to severe allergic asthma	TLR9 agonist/drug	Cytos Biotechnology AG	NCT01673672	Oligonucleotide	TH1‐mediated immune response
CYT003‐QbG10	Phase 2	Allergic Bronchial Asthma	TLR9 agonist/drug	Cytos Biotechnology AG	NCT00890734	Oligonucleotide	TH1‐mediated immune response
DUK‐CpG‐001	Phase 2	Hodgkin lymphoma, non‐Hodgkin lymphoma	TLR9 agonist/adjuvant	David Rizzieri, MD, Duke University	NCT02115126	Single‐stranded synthetic DNA molecules	Immune stimulation
MGN1703	Phases 1 and 2	HIV	TLR9 agonist/drug	University of Aarhus	NCT02443935	DNA‐based molecule	Antiviral immune stimulation
CpG‐7909	Phases 1 and 2	Non‐Hodgkin lymphoma, mycosis fungoides	TLR9 agonist/adjuvant	Ronald Levy, Lymphoma Research Foundation, American Society of Clinical Oncology, Stanford University	NCT00185965	single‐stranded synthetic DNA molecules	TH1‐like immune stimulator
CYT003	Phase 2	Asthma	TLR9 agonist/drug	Cytos Biotechnology AG	NCT02087644	Oligonucleotide	TH1‐mediated immune response
CpG‐7909 (pneumococcal vaccines)	Phases 1 and 2	HIV infections	TLR9 agonist/adjuvant	Aarhus University Hospital	NCT00562939	Oligonucleotide	Immune stimulator
GNKG168	Phase 1	Relapsed acute lymphoblastic myelogenous leukemia	TLR9 agonist/drug	Therapeutic Advances in Childhood Leukemia Consortium	NCT01743807	CpG‐C class oligodeoxynucleotide	Antitumor immune response
CpG‐7909	Phase 1	Malaria	TLR9 agonist/adjuvant	Oxford University, NIAID	NCT01351948	Oligonucleotide	Immune stimulation
EMD 1201081 (Cetuximab)	Phase 2	Squamous cell carcinoma of the head and neck	TLR9 agonist/adjuvant	EMD Serono	NCT01040832	Oligonucleotide	Immune stimulation
IMO‐2125	Phase 1	Hepatitis C	TLR9 agonist/drug	Idera Pharmaceuticals, Inc	NCT00728936	Oligonucleotide	Immune stimulation
CpG 10104	Phase 1	Hookworm infection	TLR9 agonist/adjuvant	Baylor College of Medicine, George Washington University	NCT02143518	Oligonucleotide	Immune stimulation
AZD1419	Phase 2	Asthma	TLR9 agonist/drug	AstraZeneca	NCT02898662	C‐type CpG Oligonucleotide	IFN induction
CpG‐7909 (URLC10‐177, TTK‐567)	Phases 1 and 2	Esophageal cancer	TLR9 agonist/adjuvant	Wakayama Medical University, Human Genome Center University of Tokyo	NCT00669292	Oligonucleotide	Immune stimulation
CpG‐7909	Phases 1 and 2	Mycosis fungoides	TLR9 agonist/adjuvant	Stanford University, NIH	NCT00226993	Oligonucleotide	Immune stimulation
SD‐101	Phase 1	Chronic hepatitis C	TLR9 agonist/adjuvant	Dynavax Technologies Corporation, Synteract, Inc, PPD	NCT00823862	CpG‐C class oligodeoxynucleotide	Anti0tumor immune response
Mycobacterium w.(Mw)‐freeze dried extract 0.5 mL	NA	Optic neuritis	TLR9 antagonist/adjuvant	Sudhalkar Eye Hospital	NCT01424735	Bacterial mix	Immune suppression
Hydroxychloroquine sulfate, valsartan	Phase 4	Primary IgA nephropathy	TLR9 inhibitor/adjuvant	Peking Union Medical College Hospital	NCT02765594	SM	Impaired IFN‐α and TNF‐α secretion
Hydroxychloroquine	Phase 3	Sjogren's syndrome	TLR9 inhibitor/drug	SNU Hospital	NCT01601028	SM	Immune suppression
CpG‐ODN (K3)	Phase 1	Lung tumor	TLR9 agonist/drug	National Institute of Biomedical Innovation, Osaka University	NA	Nucleotide based	Immune stimulation

Abbreviations: IFN, interferon; NA, not applicable; NIAID, National Institute of Allergy & Infectious Diseases; NCI, National Cancer Institute; NCT, national clinical trial; NIH, National Institute of Health; SM, small molecule; SNU, Seoul National University; TLR, Toll‐like receptor; TNF, tumor necrosis factor.

Similar to other TLRs, TLR9 forms a symmetrical complex with CpG‐DNA; nonetheless, during inhibitory DNA interactions, it remains in a monomeric form. CpG‐DNA binding with TLR9 is symmetric and they form a stoichiometric complex of 2:2, as DNA is recognized by both TLR9 monomers, particularly via the amino‐terminal fragment (LRRNT–LRR10) from one protomer and the carboxy‐terminal fragment (LRR20–LRR22) from the other.^142^ CpG‐DNA‐based TLR9 inhibition is mediated by binding to the concave surface formed by LRR2–LRR10, thereby inhibiting its signaling.

### 6.7 TLR10–13

Other than TLR1–9, humans also have TLR10 and TLR11, whereas they lack TLR12 and TLR13.^143^ The expression of TLR10 has been confirmed in humans (spleen, lymph node, B cells, monocytes, and neutrophils)^144^; nonetheless, its function and specific ligand are yet to be determined. Recently, it was suggested that TLR10 may act as an anti‐inflammatory TLR, rather than a conventional inflammatory receptor and that it modulates TLR2‐mediated responses through the formation of heterodimers with TLR1 or TLR6.^145^ Humans have a pseudogene homologous to TLR11 that includes a premature stop codon, resulting in lack of protein expression.^146^ TLR11 and TLR12 have been studied in mouse and they have shown to detect profilin from *Toxoplasma gondii* and be capable of forming heterodimers.^143^

## 7 INTERDEPENDENT AND CROSS‐TALK AMONG TLR PATHWAYS

Since TLRs overlap in their structures and signaling pathways, it is rational to assume that one single ligand can activate multiple TLRs; however, this is less common among plasma membrane expressed TLRs, TLR2/1, TLR2/6, TLR4, and TLR5, and there are a few ligands that can share targets, particularly for TLR2 and TLR4. This situation is very common among endosomal TLRs, partly because they are all involved in sensing nucleic acids, and endosomes have a specific pH range that is also thought to contribute to their activation. Various ligands exert their actions on multiple endosomal TLRs (eg, TLR7/8 or TLR7/8/9), which may imply a combination of multiple pathways in their activity, a common mode of activation, and, to some extent, H^+^ interference of these TLRs being a common factor^147^, ^148^ (Tables 7 and 10). TLR7 and TLR8 detect ssRNA, which may explain why one ligand is equally effective against both TLRs. Some studies have also explored the independent targeting for either TLR7^149^ or TLR8.^150^ The expression patterns of various TLRs differ among tissues, and the extent to which they respond to various ligands may contribute to unexpected results of clinical trials (mostly failure and toxicity issues). TLR2/TLR4 (cell surface) can be regulated by a single ligand, and there are many examples of endosomal TLRs being regulated by single ligands, suggesting that they may have similar sensing and regulatory mechanisms that could be exploited for therapeutic purposes.

**Table 10:** Ligands with either undefined or multiple target TLRs

Ligand	Phase	Application	Mode of action adjuvant/drug	Sponsor/collaborators	NCT number	Type	Purpose/mechanism
DC‐based vaccine	Phases 1 and 2	Melanoma	TLR agonist/adjuvant	Radboud University	NCT01530698	Biologics, SM	DC activation
TLR agonist (Tuberculin nasal challenge, timothy grass pollen)	NA	Allergic rhinitis, asthma, latent tuberculosis	TLR agonist/adjuvant	Imperial College London	NCT02090374	Bacterial mix, SM	Immune stimulation
Mycobacterium w	Phases 2 and 3	Sepsis	TLR agonist/adjuvant	Postgraduate Institute of Medical Education & Research, PGIMS, Rohtak, St. John's National Academy of Health Sciences	NCT02330432	Bacterial mix	Immune stimulation
DRibbles vaccine, imiquimod, HPV vaccine	Phase 2	Carcinoma, non‐small‐cell lung cancer	TLR2/3/4/7/9 agonists/adjuvant	UbiVac	NCT01909752	Combination of protein and SM	Immune stimulation
DRibbles vaccine, imiquimod, HPV vaccine	Phase 1	Adenocarcinoma of the prostate	TLR agonist/adjuvant	UbiVac	NCT02234921	Combination of protein and SM	Immune stimulation
MB‐11040	Phase 1	Menopause, autoimmune disease, cancer	TLR agonist/drug	KT & G Life Sciences Corp	NA	SM	Antitumor immunity
Mycobacterium w	Phases 2 and 3	Severe sepsis, septic shock, immune modulation	TLR agonist/drug	Postgraduate Institute of Medical Education & Research	NCT02025660	Bacterial mix	Immune stimulation
Insulin	Phase 2	Insulin resistance	TLR inhibitor/drug	Kaleida Health, American Diabetes Association	NCT01151605	Biomolecule	TLR downregulation
Autologous DC vaccination	Phases 1 and 2	Melanoma	TLRs agonist/adjuvant	Radboud University	NCT00940004	SM	DC activation/maturation
R848 (GP100, MAGE‐3)	Phase 2	Melanoma	TLR agonist/adjuvant	MD Anderson Cancer Center	NCT00960752	SM	Immune stimulation
VB‐201	Phase 2	Ulcerative colitis	TLR2‐4 antagonist/drug	VBL Therapeutics	NCT01839214	SM	Immune suppression
PUL‐042	Phase 1	Healthy individuals	TLR2/6, 9 agonist/adjuvant	Pulmotect Inc	NCT02124278	SM	Immune stimulation
PUL‐042	Phase 1	Hematologic diseases, stem cell transplants	TLR2/6, 9 agonist/adjuvant	Pulmotect Inc	NCT03097796	SM	Immune stimulation

Abbreviations: DC, dendritic cell; HPV, human papilloma virus; NA, not available; NCI, National Cancer Institute; NCT, national clinical trial; PGIMS, Pandit Bhagwat Dayal Sharma; SM, small molecule; TLR, Toll‐like receptor.

TLRs may antagonize one another under certain physiological conditions. For example, TLR2 and TLR9 antagonize each other in a mouse model for oral infection with *Salmonella enterica*.^151^ TLR9 deficiency is manifested as reduced survival, exaggerated cytokine responses, and salmonella hepatitis, while TLR2 deficiency produces the opposite effects. Deficiency of either TLR may disrupt NK cell cytotoxicity, and IFN‐γ and ROS production.^151^

Synergism is very common in TLRs. When monocyte‐derived DCs have been triggered with a TLR8 ligand, TLR3 or TLR4 are also activated, resulting in expression of IL‐6, IL‐10, IL‐12, and TNF‐α elevation. These results were also confirmed by increased binding of IRF and signal transducers and activators of transcription (STAT) transcription factors to their respective DNA binding sites, which was abolished when NF‐κB, p38, and phosphoinositide 3‐kinase (PI3K) inhibitors were used.^152^ These data suggest that co‐operation among TLRs is perpetuated, not only at the top level but also among different signaling pathways to ensure proper and balanced expressions of target genes.

Synergy and tolerance of TLRs are long‐established and are critical to the innate immune response. The coadministration of LPS (TLR4 agonist) and MALP‐2 (TLR2 agonist) to mouse macrophages resulted in increased TNF‐α production.^153^ Repeated treatment with LPS or MALP‐2 resulted in a hyporesponse, also termed tolerance. Intriguingly, pretreatment with any ligand results in lower responses on exposure to the second ligand.^153^ LPS may cause downregulation of the cell surface expression of TLR4 after the second LPS treatment; however, MALP‐2‐mediated reduction in responses involve modulation of downstream signaling. The acute immune tolerance and cross‐tolerance between TLR4 and TLR9 have been studied,^154^ indicating that LPS selectively inhibits proinflammatory cytokines, while CpG suppresses both pro‐ and anti‐inflammatory responses. IRAK‐M is critical for the induction of this differential response, and its expression is modulated by IL‐7.^154^

The mycobacterium extract,^155^, ^156^ and autophagosome‐enriched cancer vaccine (DRibbles),^157^ which likely contain multiple biological molecules and can trigger numerous TLRs, is being evaluated in clinical trials; however, caution is required when considering the use of such substances in the clinic due to synergy and differential responsiveness of TLRs to various ligands. Moreover, DC vaccines that have been matured using TLR ligands are also therapeutically relevant, owing to the use of TLR ligands in their production.^158^, ^159^

## 8 FAILED CLINICAL TRIALS

The proportion of failures of clinical trials depends on the clinical stage, as well as the type of disease; particularly, failure at phase 3 is an impediment to the development of successful therapy for various diseases and TLRs are no exception. For example, eritoran, a TLR4 antagonist, that was being evaluated for treatment of sepsis could not meet its target end‐point in phase 3 when data from ~2000 patients were analyzed.^160^ Among the reasons of failure of eritoran, there were oversights in study design, patient population differences, improved patient care methods, and mixed bacterial infections.^160^ Similarly, imiquimod, a TLR7 agonist, produced a divergent result in phase 3 when evaluated for treatment of the skin disorder, molluscum contagiosum (MC) lesions, in children.^161^ Imiquimod was first approved by the Food and Drug Administration in 1997 for treatment of genital warts. This approval has prejudiced its subsequent off‐label use as the treatment of MC in children, since it was already shown to be effective against viral‐based diseases and its use is supported by several research and clinical investigations.^82^, ^83^ This off‐labeled use of imiquimod is still debatable.^83^, ^161^ Similarly, in a meta‐analysis, Qin et al (162) has systematically analyzed the TLR9 agonist effects as observed by other clinical investigations. It has been concluded that the safety profile of TLR9 agonists is acceptable if they are not combine with immunosuppressive drugs.^162^

The success rate of transition among different clinical phases (phase 1, 2, 3, and occasionally 4) is highly variable. The likelihood of a molecule passing phase 1 is 63.2%, which is the highest probability for any phase. Phase 2 has the lowest success rate (30.7%), while phase 3 has a success rate of 58.1%.^163^, ^164^ Biologics have twice the final success rate (18%), compared with that of small molecules (9%). The transition failure can comprise of drugs that have not met their specific endpoints, and there are cases where particular drugs did not show any improved effect over an existing treatment of a particular condition. The situation is exacerbated when similar molecules are evaluated in multiple interventions, causing an elevated number of failed trials.

Lack of recruitment (23%) and unstated reasons (such as unknown reasons for termination, unable to begin the study, unavailability of a drug, or protocol modification resulting in cessation of a trial; 26%) comprise the majority of reasons for failure of clinical trials targeting TLRs, followed by safety issues (18%) and financial concerns (where a sponsor withdraws the drug; 15%). Moreover, 15% of trials do not show any efficacy in subsequent clinical trials. The inadequate understanding of the biology of TLRs may also contribute to drug failure,^160^ underlining the need for further studies, increased understanding of the theoretical background of disease etiology and progression, modification of protocols to address problems, and trial redesign.

While many factors contribute to a failed clinical trial, a common reason underlying failure is a lack of serious focus on biomarker discovery and implementation. There is a clear trend of success among those trials including biomarker selection (25.9%) compared with those lacking selection biomarkers (8.4%).^164^ There are several methods that can be used to reduce failure rates, such as early identification of false drug candidates, stratification of patients, development of diagnostics, proper use of pharmacogenomics through machine learning, and other analysis tools that can provide improvements and efficiencies in patient categorization. Focusing on neglected disease areas can also help to reduce the failure burden. In recent years, the Drugs for Neglected Diseases Initiative has approved six treatments within a decade, with many more in the pipeline. This is not only dramatically reducing the cost of drug development, but also providing hope for individuals affected by neglected diseases and incentivizing the pharmaceutical industry to continue their search for new drugs.

Financial and commercial reasons are also major contributors to trial failures because sponsors are “unwilling,” or there are “failure to pursue” investigational drugs for commercially important diseases. This can be reduced if pharmaceutical companies focus on diseases that lack adequate therapeutic intervention, as drugs that show positive effects will soon be marketable. Additionally, if such a trial does fail, it has a lower cost impact on the company.

Drug development is a lengthy process that starts with lead molecule identification and progresses through optimization, animal modeling studies, pharmacokinetic and pharmacodynamic studies, and preclinical and clinical stage trials. Therefore, if a drug fails to show any effect or shows toxicity in clinical studies, there must have been a series of oversights during earlier experimental stages. It is hard to give a single reason for any failure and failures may encompass complex issues, such as the use of subjective, composite, or surrogate endpoints.^165^ Moreover, biases in outcome reporting and publications; underreporting of adverse events; failure to select an appropriate patient group; preference for relative outcomes, rather than absolute values; no defined core outcome sets; lack of transparency and basic science; inappropriate study population size; and lack of data integrity are among the reasons for trial failures. Finally, during clinical trials involving humans, factors that influence the drug metabolism, distribution, and secretion are diverse that predispose the pharmacokinetically and pharmacodynamically optimized drug molecules to failure.

## 9 PERSPECTIVES IN TLR TARGETING

Researchers are expending extensive efforts to generate appropriate solutions for various inflammatory, autoimmune, and malignant conditions; however, the process is not straightforward, rather it is littered with unexpected events and outcomes, along with unknown obstructions that severely undermine the efforts of the research community.

In the majority of studies targeting TLRs, the investigated compounds are related to or derived from natural ligands; particularly those targeting TLR3, TLR4, TLR5, and TLR9, and somewhat those for TLR2. TLR7 and TLR8 have the benefit of being targeted by small molecules rather than ssRNA. The instability of ssRNA molecules can hinder their use for TLRs activation. However, since RNAi technology is being evaluated in more than 100 clinical trials,^166^ stability should not be an issue, rather, tuning of small molecules is far easier than tuning biologics for therapeutic purposes.

Other than molecules derived from natural ligands, it is necessary to focus on the chemical space that can be used to target TLRs.^106^, ^118^ This broadening of the chemical space will provide more potent, specific, and less toxic molecules, resulting in fewer trial failures. Biologics are gaining popularity, as they have a higher ratio of success, and are comparatively safe and specific.^167^, ^168^, ^169^, ^170^, ^171^ It is estimated that the biologics will soon become the norm in therapeutics, in addition to being responsible for the majority of revenue.^172^ For different TLRs, the therapeutic trend can vary; however, a rise in antibody‐mediated TLR inhibition (TLR2, TLR3, and TLR4) and novel molecular backbone (independent of PAMPs) have been seen in recent therapeutics.

The evaluation of various drugs for similar or different conditions is also an optimal approach, which can facilitate the development of single drugs for multiple diseases. In this context, research laboratories can screen the outcomes of phase 2 failures that have been abandoned by their sponsors to evaluate them for other symptoms.^173^ Such an approach can dramatically reduce the cost, speed up the process, and will encourage pharmaceutical companies to share their data with research laboratories for application to other disease targets.

Rather than directly inhibiting TLRs, it may be more appropriate to target the transcription regulation of TLRs to suppress their expression,^174^ as described in a study where the authors used GST‐21 for cytokine inhibition, which could be reversed by the janus kinase 2 inhibitor, AG490. Since the majority of TLRs regulate the similar pathway, targeting of their downstream inhibitory signaling mechanisms should also be explored to further intensify the benefits of their inhibition.

Lack of clinical data is an impediment to the development of clinical research. It is estimated that approximately half of all clinical trials are not reported in either peer‐reviewed journals or clinical trial websites (clinicaltrials.gov; http://apps.who.int/trialsearch/).^175^, ^176^

It is now necessary to develop additional TLR ligands that should not mimic PAMPs, explore new biomarkers for disease progression, revise protocols, and clinical trials, target small subsets of patients, improve the understanding of the basic biology of diseases, and improve final outcomes, which must legitimately refer to the progress of the disease and the effect of the compound being applied.

## 10 CONCLUSIONS

TLRs are among the ideal targets for exploitation in immunotherapy; however, their biology still needs to be better understood in the context of target diseases. These receptors are capable of inhibiting disease pathophysiology, as well as exacerbating inflammatory diseases. Given this dual role, it is imperative to fine tune their activation using a multidrug approach. Cumulative evidence suggests the participation of TLRs in almost all diseases is unique and can be exploited by including their ligands as adjuvant treatment during regular immunotherapy or as part of other therapeutic regimens.

It is vital to create superior disease models that assist in early phase evaluation of drugs, improve diagnostics and evaluation of disease progression, and facilitate identification of novel biomarkers that reliably indicate disease progression and real‐time disease monitoring. Finally, the availability of clinical trial data should be ensured to guide the scientific community in their endeavors. This would also assist in the refinement of targets and lead molecules and improve the pathophysiological manifestations of diseases. Using a combination of computational power, next‐generation sequencing and proteomic data, machine learning approaches, and improved availability of results, we are hopeful that a dramatic increase in new therapeutic options for various inflammatory diseases and cancers involving TLRs and a decline in clinical trial failures will be achieved.

## CONFLICTS OF INTEREST

The authors declare that there are no conflicts of interest.

## AUTHOR'S CONTRIBUTION

MAA and SC participated in research design; MAA, MS, and JK performed data analysis; and MAA, MS, JK, and SC wrote or contributed to the writing of the manuscript.
